# Supplementary figures and images for: Selection of genotypes harbouring mutations in the cytochrome b gene of Theileria annulata is associated with resistance to buparvaquone
Source: PLoS One. 2023 Jan 4;18(1):e0279925. doi: 10.1371/journal.pone.0279925 (PMC9812330; doi:10.1371/journal.pone.0279925)

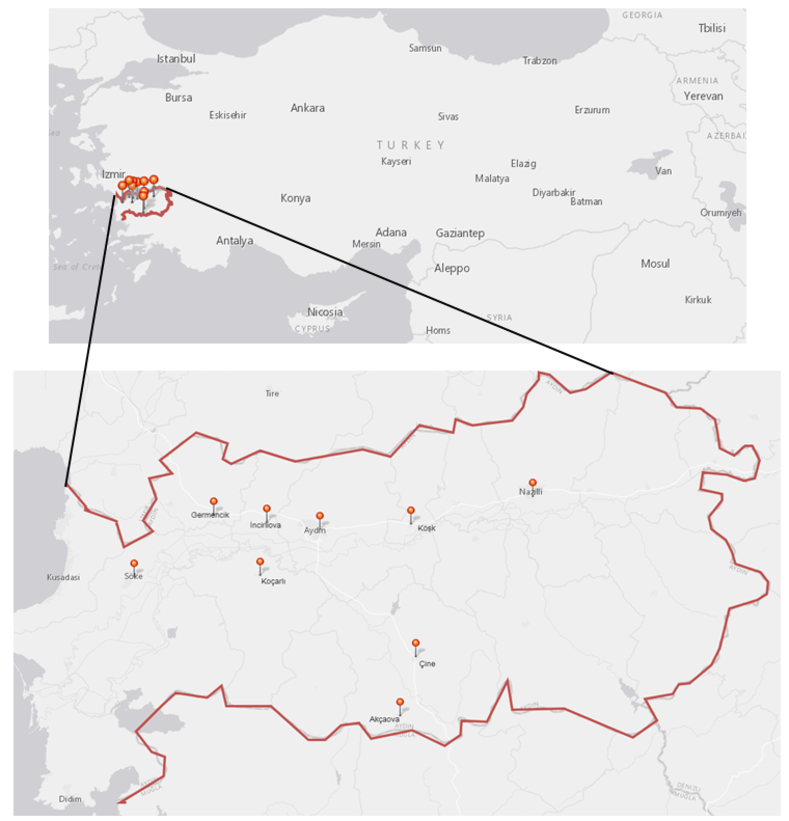

Supplement: S1 Fig — The geographical distribution of the provinces where the parasite material used in this study was obtained. The map was prepared using the USGS National Map Viewer (public domain): http://viewer.nationalmap.gov/viewer/ with some modification. (TIF) [file pone.0279925.s001.tif]

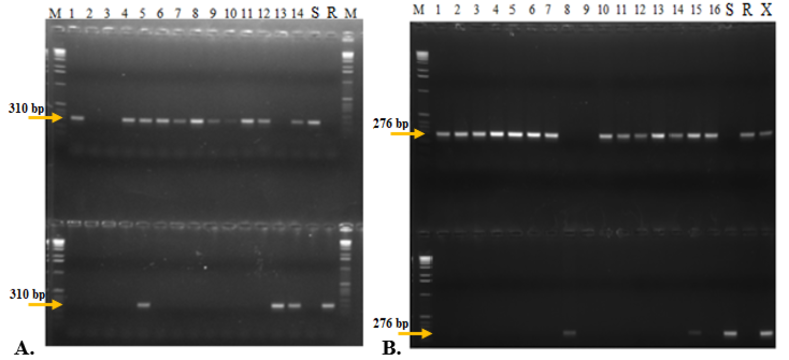

Supplement: S2 Fig — Gels showing detection of PCR amplicons of isolates with mutations V135A (A) and P253S (B), respectively. Products amplified using drug sensitive and resistance specific forward primers are given at the top and bottom of each gel, respectively. M, 100 bp molecular size marker (Thermo Scientific Corp.); lanes 1–16, products from template DNA of T. annulata samples; lanes S sensitive control, lanes R resistance control, lane X mixed control. (TIF) [file pone.0279925.s002.tif]
