# Supplementary material for: Selection of genotypes harbouring mutations in the cytochrome b gene of Theileria annulata is associated with resistance to buparvaquone
Source: PLoS One. 2023 Jan 4;18(1):e0279925. doi: 10.1371/journal.pone.0279925 (PMC9812330; doi:10.1371/journal.pone.0279925)
Supplement: S1 Table — (PDF) [file pone.0279925.s003.pdf]

S1 Table. IC_{50} values of different *T. annulata* isolates prepared before and after buparvaquone treatment.

Animal Code	Isolate Code	Treatment	IC_{50} (ng/mL)
**1**	A1/BT	Before treatment	2.0
**2**	A2/BT	Before treatment	3.20
**3**	A3/BT	Before treatment	2.63
**4**	A5/BT	Before treatment	2.16
**5**	A6/BT	Before treatment	3.52
**6**	A7/BT	Before treatment	6.95
**7**	A8/BT	Before treatment	6.59
**8**	A9/BT	Before treatment	0.72
**9**	A10/BT	Before treatment	16.75
	A10/AT3	After 3rd treatment	135.0
**10**	A11/AT1	After 1st treatment	1.83
**11**	A12/AT1	After 1st treatment	2.96
**12**	A13/AT1	After 1st treatment	3.83
**13**	A14/AT2	After 2nd treatment	2.62
**14**	A15/AT3	After 3rd treatment	0.63
**15**	A16/AT1	After 1st treatment	5.56
**16**	A17/AT1	After 1st treatment	5.0
**17**	A18/AT1	After 1st treatment	3.63
**18**	A21/AT1	After 1st treatment	16.75
	A21/AT3	After 3rd treatment	25.53
	A21/AT4	After 4th treatment	73.79
**19**	A24/BT	Before treatment	1.95
**20**	A25/AT1	After 1st treatment	1.46
**21**	A26/BT	Before treatment	3.27
**22**	A27/BT	Before treatment	1.40
**23**	A28/BT	Before treatment	1.47
**24**	A29/BT	Before treatment	1.39
**25**	A30/BT	Before treatment	1.19
**26**	A31/BT	Before treatment	2.58

Animal Code	Isolate code	Treatment	IC_{50} (ng/mL)
**43**	A115/AT1	After 1st treatment	3.17
	A115/AT2	After 2nd treatment	6.36
**44**	A116/AT2	After 2nd treatment	5.02
**45**	A117/AT1	After 1st treatment	2.16
**46**	A118/BT	Before treatment	5.02
**47**	A119/BT	Before treatment	2.27
**48**	A120/AT1	After 1st treatment	2.18
**49**	A121/AT1	After 1st treatment	2.05
**50**	A122/AT1	After 1st treatment	9.66
	A122/AT2	After 2nd treatment	10.70
**51**	A123/BT	Before treatment	3.87
**52**	A124/AT1	After 1st treatment	1.34
**53**	A125/BT	Before treatment	2.91
**54**	A126/BT	Before treatment	1.34
**55**	A127/BT	Before treatment	1.15
**56**	A128/BT	Before treatment	0.69
**57**	A129/BT	Before treatment	1.67
**58**	A130/BT	Before treatment	1.54
**59**	A131/BT	Before treatment	0.34
**60**	A132/AT2	After 2nd treatment	3.21
**61**	A133/BT	Before treatment	1.86
**62**	A134/AT1	After 1st treatment	3.28
**63**	A135/BT	Before treatment	0.85
**64**	A136/BT	Before treatment	2.02
**65**	A137/BT	Before treatment	0.52
**66**	A138/BT	Before treatment	0.73
**67**	A139/BT	Before treatment	1.02
**68**	A143/AT1	After 1st treatment	2.31

Animal Code	Isolate code	Treatment	IC_{50} (ng/mL)
**85**	A162/BT	Before treatment	1.34
**86**	A163/BT	Before treatment	1.96
**87**	A164/BT	Before treatment	0.88
**88**	A166/BT	Before treatment	2.57
**89**	A167/BT	Before treatment	2.09
**90**	A168/AT1	After 1st treatment	3.27
	A168/AT3	After 3rd treatment	4.59
**91**	A169/BT	Before treatment	0.68
**92**	A170/AT1	After 1st treatment	3.21
**93**	A171/BT	Before treatment	0.76
**94**	A172/BT	Before treatment	0.79
**95**	A173/BT	Before treatment	0.24
**96**	A174/BT	Before treatment	2.27
**97**	A175/BT	Before treatment	2.0
**98**	A176/BT	Before treatment	3.21
**99**	A177/AT1	After 1st treatment	2.97
	A177/AT2	After 2nd treatment	6.36
**100**	A178/BT	Before treatment	2.63
**101**	A179/BT	Before treatment	2.16
	A179/AT1	After 1st tretment	1.97
**102**	A180/BT	Before treatment	1.47
	A180/AT2	After 2nd treatment	7.48
**103**	A181/BT	Before treatment	1.95
**104**	AC1/BT	Before treatment	4.36
**105**	AC2/BT	Before treatment	1.34
**106**	G1/BT	Before treatment	1.46
**107**	G2/BT	Before treatment	2.05
**108**	G3/BT	Before treatment	4.73
**109**	G4/BT	Before treatment	1.57
**110**	N1/BT	Before treatment	2.31

**27**	A32/BT	Before treatment	1.15	**69**	A144/BT	Before treatment	0.85	**111**	N2/BT	Before treatment	1.98
**28**	A33/BT	Before treatment	1.34	**70**	A145/AT2	After 2nd treatment	1.67	**112**	N3/BT	Before treatment	3.15
**29**	A34/BT	Before treatment	0.76	**71**	A148/AT4	After 4th treatment	6.20	**113**	N4/BT	Before treatment	2.31
**30**	A100/BT	Before treatment	2.16	**72**	A149/BT	Before treatment	1.50	**114**	N5/BT	Before treatment	2.04
	A100/AT1	After 1st treatment	1.51								
**31**	A101/BT	Before treatment	2.07	**73**	A150/AT1	After 1st treatment	2.97	**115**	K1/BT	Before treatment	4.69
	A101/AT2	After 2nd treatment	1.50								
**32**	A102/BT	Before treatment	3.52	**74**	A151/AT1	After 1st treatment	6.36	**116**	K2/BT	Before treatment	3.87
**33**	A103/AT1	After 1st treatment	1.57	**75**	A152/BT	Before treatment	1.69	**117**	M1/BT	Before treatment	3.19
**34**	A106/BT	Before treatment	2.58	**76**	A153/BT	Before treatment	0.92	**118**	M2/AT1	After 1st treatment	0.80
**35**	A107/BT	Before treatment	1.15	**77**	A154/AT1	After 1st treatment	1.15	**119**	M3/BT	Before treatment	2.91
**36**	A108/BT	Before treatment	1.34	**78**	A155/AT1	After 1st treatment	3.11	**120**	M4/BT	Before treatment	4.59
**37**	A109/BT	Before treatment	5.56	**79**	A156/AT1	After 1st treatment	2.16	**121**	CN1/BT	Before treatment	0.78
	A109/AT2	After 2nd treatment	1.55		A156/AT2	After 2nd treatment	3.52				
					A156/AT3	After 3rd treatment	4.73				
**38**	A110/AT3	After 3rd treatment	5.56		A157/BT	Before treatment	1.55	**122**	CN2/BT	Before treatment	1.39
**39**	A111/BT	Before treatment	1.83	**81**	A158/BT	Before treatment	0.89	**123**	CN3/BT	Before treatment	3.56
**40**	A112/BT	Before treatment	1.28	**82**	A159/BT	Before treatment	0.69	**124**	C1/BT	Before treatment	1.37
**41**	A113/BT	Before treatment	3.19	**83**	A160/BT	Before treatment	1.67	**125**	S1/AT1	After 1st treatment	2.57
**42**	A114/AT1	After 1st treatment	1.34	**84**	A161/BT	Before treatment	1.57	**126**	S2/AT1	After 1st treatment	2.71

The letter at the beginning of the isolate code represents the provinces from which the isolate originated (A; Akcaova, AC; İncirliova, G; Germencik, N; Nazilli, K; Kösk, M; Center, CN; Koçarlı, C;Cine, S; Söke).
BT; indicates *T. annulata* isolates prepared before drug treatment
AT; indicates *T. annulata* isolates prepared after drug treatment. AT1-4 represents the number of drug treatments.
