# Supplementary material for: Selection of genotypes harbouring mutations in the cytochrome b gene of Theileria annulata is associated with resistance to buparvaquone
Source: PLoS One. 2023 Jan 4;18(1):e0279925. doi: 10.1371/journal.pone.0279925 (PMC9812330; doi:10.1371/journal.pone.0279925)
Supplement: S2 Table — (PDF) [file pone.0279925.s004.pdf]

S2 Table. Summary of mutations detected in *TaPIN1* sequences of *T. annulata* isolates from Tunisia, Sudan and Turkey.

	**Amino acid position**	**1**	**2**	**22**	**23**	**24**	**26**	**53**	**61**	**78**	**96**	**143**
	TaPIN1 TA18945	M	I	T	I	I	A	A	A	L	R	STOP
**Tunisian isolate**	Bup. resistant *TaPIN1*	-	-	I	-	-	L	P	-	-	-	-
**Sudanese isolates**	16 isolate	L	-	-	-	-	-	-	-	-	-	-
	Th212	-	-	A	-	-	-	-	-	-	-	-
	Th10,	-	-	-	N	-	L	-	-	-	-	-
	E21	-	-	-	N	-	P	-	-	-	-	-
	E1, Th210	-	-	-	-	V		-	-	-	-	-
	21 isolate	-	-	-	-	-	P	-	-	-	-	-
	B241	-	-	-	-	V	-	P	-	-	-	-
	E23	-	-	-	-	-	-	-	G	-	-	Y
	Th18	-	-	-	-	-	-	-	-	-	-	Y
**Turkish isolates**	A21/AT4/cl-3, 8	-	F	I	-	-	T	-	-	-	-	-
	A10/AT3/cl-8 , 9	-	-	-	-	-	-	-	-	-	-	-
	A9/BT/cl-5	-	-	A	N	-	P	-	-	-	-	-
	G3/BT/cl-2	-	-	-	-	-	-	-	-	-	K	-
	N3/BT/cl-4	-	-	-	-	-	-	-	-	P	-	-
