# Supplementary material for: Selection of genotypes harbouring mutations in the cytochrome b gene of Theileria annulata is associated with resistance to buparvaquone
Source: PLoS One. 2023 Jan 4;18(1):e0279925. doi: 10.1371/journal.pone.0279925 (PMC9812330; doi:10.1371/journal.pone.0279925)
Supplement: S3 Table — (PDF) [file pone.0279925.s005.pdf]

S3 Table. AS-PCR results of clonal cell lines.

Isolate code	Number of clonal cell lines (n)	Presence of Mutations			
		with		without	
		V135A	P253S	V135A	P253S
**G3/BT**	39	-	-	39	39
**N3/BT**	42	-	-	42	42
**A9/BT**	45	-	-	45	45
**A10/BT**	41	38	-	3	41
**A10/AT3**	45	45	-	-	45
**A16/AT1**	40	-	-	40	40
**A21/BT**	48	-	48	48	-
**A21/AT4**	49	-	8	49	41
