# Supplementary material for: Selection of genotypes harbouring mutations in the cytochrome b gene of Theileria annulata is associated with resistance to buparvaquone
Source: PLoS One. 2023 Jan 4;18(1):e0279925. doi: 10.1371/journal.pone.0279925 (PMC9812330; doi:10.1371/journal.pone.0279925)
Supplement: S4 Table — (PDF) [file pone.0279925.s006.pdf]

S4 Table. Summary of mutations detected in *Cyto b* sequences of *T. annulata* isolates from Tunisia, Sudan, Iran and Turkey.

**Nucleotide**	**234**	**385**	**404**	**417**	**429**	**436**	**679**	**757**	**870**
**Codon**	**78**	**129**	**135***	**139**	**143**	**146***	**227***	**253***	**290**
Tancytb (XM949625)	TCG	AGC	GTC (Val)	TTA	TTC	GCT (Ala)	GTG (Val)	CCT (Pro)	GTA
Position of common mutations	..A	G..	.C. (Ala)	..G	..T	A.. (Thr)	A.. (Met)	T.. (Ser)	..G
Tunisian samples		+		+	+	+		+	+
Sudanese samples	+	+		+	+	+	+		+
Iranian samples		+						+	
Egyptian samples								+	
Turkish samples	+		+	+		+	+	+	+

(*) indicates the non-synonymous mutations sites
