# Supplementary material for: Selection of genotypes harbouring mutations in the cytochrome b gene of Theileria annulata is associated with resistance to buparvaquone
Source: PLoS One. 2023 Jan 4;18(1):e0279925. doi: 10.1371/journal.pone.0279925 (PMC9812330; doi:10.1371/journal.pone.0279925)
Supplement: Attachment — Submitted filename: Response to Reviewers.docx [file pone.0279925.s008.docx]

Dear Dr. Vikrant Sudan,

Thank you for your response to our manuscript PONE-D-22-20200R1 entitled " Selection of genotypes harbouring mutations in the cytochrome b gene of *Theileria annulata* is associated with resistance to buparvaquone". We would also like to thank both the reviewers for their comments to improve the quality of the manuscript.

The figures of the manuscript were uploaded to the PACE before resubmitting the manuscript and the corrected version of the figures by PACE were submitted to PLOS ONE. On the following pages, we present our point-by-point responses to the comments and suggestions made by the Academic Editor and reviewers.

A revised version of our manuscript is also provided, where all modifications are shown in a separate file labeled 'Revised Manuscript with Track Changes’. Please be informed that all the changes made in the text as a response to reviewers are recorded using Track Changes in MS Word and highlighted in yellow collor.

Yours sincerely,

Tulin KARAGENC (DVM, PhD)

**Response to Journal Requirements:**

**Comment:** Please review your reference list to ensure that it is complete and correct. If you have cited papers that have been retracted, please include the rationale for doing so in the manuscript text, or remove these references and replace them with relevant current references. Any changes to the reference list should be mentioned in the rebuttal letter that accompanies your revised manuscript. If you need to cite a retracted article, indicate the article’s retracted status in the References list and also include a citation and full reference for the retraction notice.

The reference list has been checked and corrected according to the journal style. It is complete and we did not cite any papers that have been retracted.

**Response to Reviewers' comments:**

**Response to Reviewer 2:**

**Minor points**

**Comment**. Line 47-48: “The obtained data strongly suggest that the genetic mutations resulting in V135A and P253S detected at the putative binding sites of buparvaquone in cytochrome b play a significant role in conferring,” may be written as “The observed data strongly suggested -----------”

Beginning of the sentence has been re-written as follows: “The observed data strongly suggested -------” **(Line 48 in the modified manuscript).**

**Comment**. Line 79: “disrupting the ubiquinone synthesis pathway”????

The sentence has been re-written as “It is also thought that buparvaquone acts as a peptidyl-prolyl isomerase PIN1 inhibitor**.” (Line 79 in the modified manuscript)**

**Comment**. Line147, 149, 239, 243, 365: μl maybe written as μL

All “μl” has been replaced by ‘μL’ in the text.

**Comment**. Line 281: demonstrated

The word **“**demonstrates” has been replaced by “demonstrated”. **(Line 281 in the modified manuscript)**

**Comment**. Line 307: of buparvaquone resistance

The preposition “of” has been added before the “buparvaquone resistance” **(Lines 306-307 in the modified manuscript).**
